# Persistent structures in a three-dimensional dynamical system with flowing and non-flowing regions

**DOI:** 10.1038/s41467-018-05508-7

**Published:** 2018-08-07

**Authors:** Zafir Zaman, Mengqi Yu, Paul P. Park, Julio M. Ottino, Richard M. Lueptow, Paul B. Umbanhowar

**Affiliations:** 1 Department of Chemical and Biological Engineering, Northwestern University, Evanston, IL 60208 USA; 2 Department of Engineering Sciences and Applied Mathematics, Northwestern University, Evanston, IL 60208 USA; 3 Department of Mechanical Engineering, Northwestern University, Evanston, IL 60208 USA; 4 The Northwestern Institute on Complex Systems (NICO), Northwestern University, Evanston, IL 60208 USA

## Abstract

Mixing of fluids and mixing of solids are both relatively mature fields. In contrast, mixing in systems where flowing and non-flowing regions coexist remains largely unexplored and little understood. Here we report remarkably persistent mixing and non-mixing regions in a three-dimensional dynamical system where randomness is expected. A spherical shell half-filled with dry non-cohesive particles and periodically rotated about two horizontal axes generates complex structures that vary non-trivially with the rotation angles. They result from the interplay between fluid-like mixing by stretching-and-folding, and solids mixing by cutting-and-shuffling. In the experiments, larger non-mixing regions predicted by a cutting-and-shuffling model alone can persist for a range of protocols despite the presence of stretching-and-folding flows and particle-collision-driven diffusion. By uncovering the synergy of simultaneous fluid and solid mixing, we point the way to a more fundamental understanding of advection driven mixing in materials with coexisting flowing and non-flowing regions.

## Abstract (web summary)

Understanding mixing in yield stress materials, such as paint and sand, is complicated due to the coexistence of solid-like and fluid-like regimes. Zaman et al. examine mixing in a granular material in three dimensions and find persistent complex non-mixing structures within the chaotic flowing regime.

## Introduction

The goal of mixing is to rearrange initially segregated matter into states where the constituent elements are homogeneously distributed. In fluids, where the elements are atoms or molecules, mixing at low Reynolds numbers can be achieved by the stretching-and-folding of chaotic flows combined with thermal diffusion which drives mixing at the smallest length scales^1–3^. In bulk solids composed of macroscopic (athermal) particles, the particles can be deliberately rearranged, as in the cutting-and-shuffling of a deck of cards^4^. Other rheological materials–Bingham fluids and polymer mixtures^5^ for example–fall between these two extremes.

While mixing of fluids and mixing of solids have long histories and are relatively mature fields, little is understood about mixing when flowing and non-flowing regions coexist, especially in three-dimensions (3D). For example, in yield stress materials, constituent elements move together as a solid where local stresses are low, but flow in relative motion where the yield stress is exceeded. Common examples of yield stress materials include paint, concrete paste, polymer mixtures^5^, and granular materials, e.g., sand. Understanding 3D mixing in such materials is critical in many domains where variations in local concentration can be disastrous, including the pharmaceutical industry^6^, composite materials^7^, and concrete manufacturing. Understanding when and how flowing and non-flowing regions interact in the presence of both stretching-and-folding and cutting-and-shuffling will likely lead to new and more effective mixing methods that take advantage of both mechanisms simultaneously. An amusing but noteworthy example of mixing in a system with flowing and non-flowing regions is the Spanish Christmas Lottery (the second longest continuously running lottery with the largest payout in the world), where 100,000 wooden balls are mixed within a 2 m diameter sphere rotated about a horizontal axis with the expectation of randomness^8^. Our results show that this expectation cannot be taken for granted.

To study the interaction of mixing by stretching-and-folding with mixing by cutting-and-shuffling, we consider a geometrically simple 3D model system with localized flow—a spherical tumbler half-filled with a dry granular material and rotated alternately about orthogonal horizontal axes. Experiments with this system demonstrate the existence and extraordinary persistence of non-mixing island structures due to cutting-and-shuffling for many different experimental protocols, even in the presence of diffusion and flowing regions. To understand the structure, we employ the mathematics of piecewise isometries to predict the geometric skeleton of the mixing and non-mixing regions, demonstrating that for a range of tumbling protocols, the mixing structure is a fundamental consequence of the dynamics of cutting-and-shuffling. We further utilize a continuum model that connects the solids mixing by cutting-and-shuffling with fluid-like mixing by stretching-and-folding. This model fully captures the mixing and non-mixing regions observed in the experiments and provides insight into the interaction between stretching-and-folding and the underlying structure of the dynamical system based on cutting-and-shuffling.

## Results

### Persistent periodic structures

**Fig. 1:**
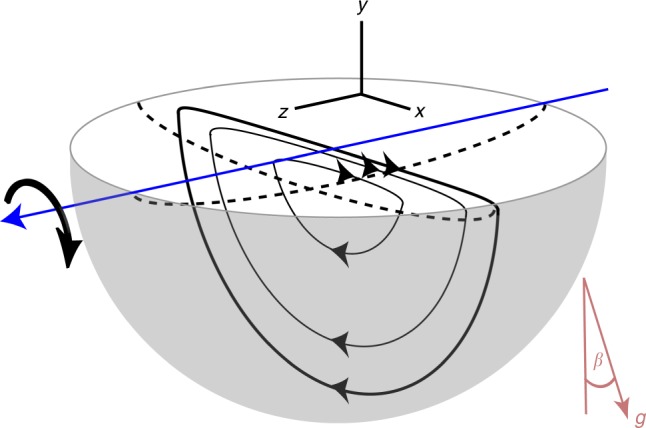
Spherical tumbler geometry and flow. Sketch showing three representative particle trajectories (curves with arrowheads) in the flowing layer and in solid body rotation in a plane normal to the free surface and perpendicular to the *z*-rotation axis. The two dashed curves show the lower boundary of the flowing layer in planes normal to the free surface and parallel and perpendicular to the rotation axis

In a tumbler, (a common device used in many industrial processes such as particle mixing, coating, and drying), particles flow in a relatively thin layer at the free surface, while below the surface in the non-flowing bed, particles move together in solid body rotation about the rotation axis, see Fig. 1. To mix the granular material in our half-filled *D*(= 2*R*) = 14 cm diameter spherical tumbler, the tumbler is rotated alternately about orthogonal horizontal axes by angles (*θ*_*z*_, *θ*_*x*_) beyond the repose angle of the granular material, *β*^9^. To characterize the mixing, we use X-ray imaging to track the location of a 4 mm diameter spherical tracer particle in a bed of 2 mm diameter glass spheres after each iteration for a wide range of protocols [i.e., (*θ*_*z*_, *θ*_*x*_) pairs], details are provided in the Methods. The larger diameter tracer particle flows on the free surface and is subsequently deposited in the bed near the tumbler wall.

**Fig. 2:**
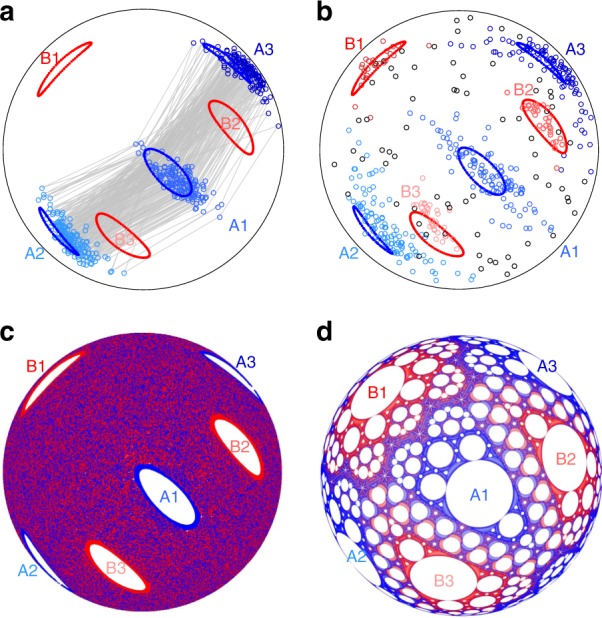
Period-3 non-mixing regions under the (57°, 57°) protocol. **a** In one experiment, the tracer particle cycles between period-3 regions A1-A3 (blue circles) with stroboscopic paths (gray lines) for 500-iterations of the protocol. **b** In a second experiment with the same macroscopic initial conditions, the tracer particle cycles between period-3 regions A1-A3 (blue circles) for 326 iterations (1–90, 96–331), period-3 regions B1-B3 (red circles) for 101 iterations (386–486), and outside of period-3 regions (black circles) for 73 iterations (91–95, 332–385, and 487–500). **c** Passive tracers in the finite flowing layer (FL) model mix everywhere except in two sets of period-3 regions (A1-A3 and B1-B3), whose boundaries are superimposed on the experimental data in **a**, **b**. **d** The piecewise isometry model (PWI), which describes solids mixing by cutting-and-shuffling, predicts unmixed cells (white regions) of which only the largest cells, (A1-A3, B1-B3) persist in the FL model (**c**). Period-3 trapping regions from experiments correspond with period-3 cells from the FL and PWI models [blue (red) curves for regions A1-A3 (B1-B3)]. Bottom-views (+*y*-direction) shown in all panels

Using our experimental apparatus, we consider first the itinerary of the tracer particle for an example protocol when the spherical tumbler is alternately rotated by 57° about orthogonal horizontal axes [a (57°, 57°) protocol] for 500 iterations. The position of the tracer particle viewed from the bottom of the spherical tumbler after each iteration (small circles) is shown in Fig. 2a. After each iteration the tracer particle is alternately displaced between three distinct regions as indicated by the gray lines between the tracer particle positions. In this run, the tracer particle never departs from these three non-mixing regions (islands) despite collisions with other particles that drive random collisional diffusion, and other minor experimental errors including small variations in the rotation angle. These effects, however, can play a role. Figure 2b shows a second 500 iteration experiment under the same conditions for which the tracer particle moves periodically between the same three regions (blue points) but also between a second set of period-3 regions (red points) and aperiodically (black points). The aperiodic motions, where the tracer particle is not in one of the two sets of period-3 regions, occur in only 73 of the 500 iterations but allow the tracer particle to move from one set of period-3 regions (blue) to the other set (red) and back. Similar period-3 non-mixing regions are observed in the ‘nearby’ protocols (54°, 54°) and (60°, 60°).

To better understand the existence of these non-mixing and mixing regions, we first consider the predictions of a standard and simple advection based continuum model^9^ for flow in a half-filled tumbler. In the model (Methods section), particles flow down the surface in a thin flowing layer (FL) that lies on top of non-flowing particles in the bulk that move in solid body rotation with the tumbler. For rotation about any axis (the *z*-axis here with *x* in the streamwise direction and *y* normal to the free surface), the non-dimensionalized velocity field **u** = (*u*, *v*, *w*) is piecewise defined such that the FL (0 ≥ *y* ≥ −*δ*) velocity is **u**_fl_ = $((\delta + y){\mathrm{/}}\epsilon ^2,xy{\mathrm{/}}\delta ,0)$ and the bulk (*y* < −*δ*) solid body rotation velocity is **u**_b_ = (*y*, −*x*, 0). The interface of the lenticular FL with the bulk is located at *δ*(*x*, *z*) = $\epsilon \sqrt {1 - x^2 - z^2}$, where $\epsilon = \delta (0,0) = \sqrt {\omega {\mathrm{/}}\dot \gamma }$ is the maximal dimensionless FL depth at the center of the sphere (*x* = *z* = 0) for shear rate $\dot \gamma$ and angular rotation velocity *ω*. All variables are dimensionless—lengths are normalized by the tumbler radius *R* and the rotation period *T* is normalized by 1/*ω*. This FL continuum model, which includes stretching characteristic of chaotic flows^3^, is parameterized by the FL depth $\epsilon$, which is set to 0.15 to match the conditions in the experiments (Methods section).

To characterize mixing in the FL model, blue passive tracer points are seeded at the intersection of the FL boundary *δ* and a hemispherical subshell having normalized radius *r* = 0.9 before the first rotation, while red points are seeded in the same location after a half-iteration (i.e., rotation about the *z*-axis). (Radius *r* = 0.9 was used to mimic tracer particle position in experiments as elliptic regions at larger *r* > 0.9 reveal more intricate structures not apparent in experiments likely due to particle size effects and collisional diffusion. As the intricate elliptic regions at *r* > 0.9 occur at the same location on the hemispherical shell as elliptic regions at *r* = 0.9, we use the elliptic orbits at *r* = 0.9 for comparison to experiment.) In Fig. 2c, the Poincaré (stroboscopic) map (see, e.g., ref. ^10^) of tracer points advected by the FL model under the same conditions as the experiment [Fig. 2a] displays uniform mixing throughout the domain except for six empty regions. These empty regions correspond to two sets of period-3 regions (A1-A3 and B1-B3): a point initially in A1 cycles to A2 in the next iteration, to A3 in the following iteration, and then back to A1. Overlaying the non-mixing regions in Fig. 2c on the experimental tracer results in Fig. 2a, b shows that they correspond to the regions in experiments where the tracer particle lingers.

Although the FL model captures the main features of the experiments shown in Fig. 2a, b, deeper insight into why persistent period-3 structures form under the (57°, 57°) protocol (and period-*n* structures for other protocols) is gained from a perspective based exclusively on solids mixing by cutting-and-shuffling. This is accomplished by taking the $\epsilon \to 0$ limit (an infinitely thin FL). In this theoretical limit, particles instantaneously jump across the free surface to a downstream point symmetric about the midpoint of the free surface and, consequently, undergo only solid body rotation. Since there is no shear in the FL for a half-filled tumbler, the biaxial mixing protocol in the $\epsilon \to 0$ limit corresponds to a radially invariant hemispherical domain with mixing dynamics that are equivalent to slicing the hemisphere into four pieces that are rearranged and then reassembled into a hemisphere again. This type of transformation is called a piecewise isometry (PWI)^11–15^ and it has found use in several applications^16–20^. Here, and similar to the FL model, the boundaries formed by the slicing after a rotation about a single axis are used as initial conditions for tracer points whose trajectories form a subset of the exceptional set^21^, which is the skeleton for transport dynamics in PWI systems. Blue tracers are seeded where the domain is cut by the action of the *θ*_*z*_ rotation, while red tracers are seeded where the domain is cut by the *θ*_*x*_ rotation. The mixing mechanism for a PWI is simply cutting-and-shuffling in analogy with mixing a deck of cards^22,23^, but this does not prevent PWIs from possessing complicated dynamics^13–15,24,25^. In fact, the hemispherical PWI system described here has several interesting properties including non-mixing regions^26,27^, resonances corresponding to non-mixing regions^28^, and a fractal nature^29^.

Similar to the FL model, repeated iteration of the four piece PWI model for the (57°, 57°) protocol [Fig. 2d] generates open regions devoid of tracers, known as cells. These cells vary in size and periodicity across the domain, with certain areas dominated by a particular color of tracer particle. The largest circular cells have the lowest periodicity. The one-to-one correspondence in size and location between the largest cells in the PWI model (A1-A3 and B1-B3), the elliptical domains in the FL model [Fig. 2c], and the non-mixing regions in the experiments [Fig. 2a, b] is remarkable. This agreement suggests that the periodic regions observed in experiment for the (57°, 57°) protocol result from the solids mixing structure generated by cutting-and-shuffling, and that it is the cutting-and-shuffling that prescribes the underlying structure, or ‘skeleton’ of the mixing.

### Dependence on rotation protocol

**Fig. 3:**
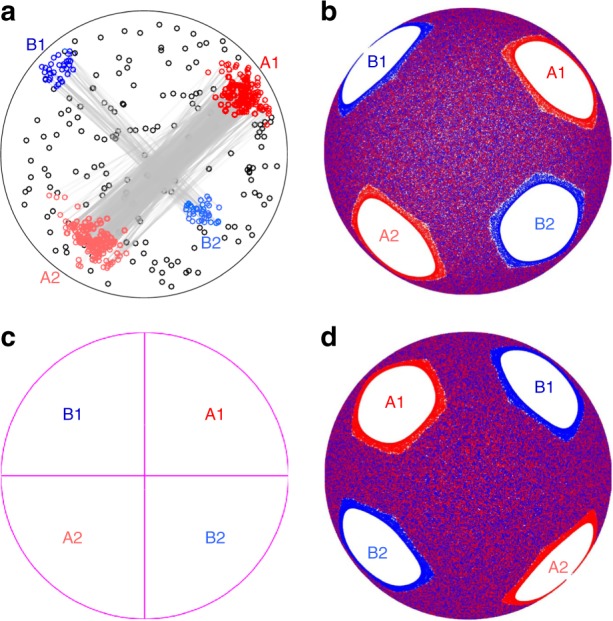
Period-2 non-mixing regions under the (90°, 90°) protocol. **a** Tracer particle in a 500-iteration experiment appears in period-2 regions A1-A2 (red circles) for 263 iterations (mean 38 iterations per instance) and B1-B2 (blue circles) for 46 iterations (mean 12 iterations per instance), stroboscopic paths indicated by gray lines. **b** In the FL model, two sets of period-2 regions (A1-A2 and B1-B2) exist. **c** The PWI model undergoes trivial period-2 rearrangement of the entire domain. **d** Interchange of non-mixing A and B regions in the FL model at 500 plus one-half-iteration illustrates the generic (i.e., protocol independent) half-period offset relationship between A and B non-mixing regions

The periodicity, as well as the size and location, of non-mixing structures depends on the rotation protocol. For example, consider the results for the (90°, 90°) protocol, which has period-2 non-mixing regions, shown in Fig. 3. In experiment [Fig. 3a], a tracer particle seeded in the A1 region, cycles between A1 and A2 and between B1 and B2 for a total of 263 and 46 iterations, respectively, for a 500-iteration experiment. Compared to the period-3 non-mixing regions under the (57°, 57°) protocol in Fig. 2, the tracer escapes more frequently from the period-2 non-mixing regions. Again, the non-mixing regions (A1-A2 and B1-B2) correspond well with the islands in the FL model [Fig. 3b], while in the PWI model [Fig. 3c] the four period-2 non-mixing regions occupy the entire domain since the entire hemisphere returns to its initial condition every six iterations. As with the (57°, 57°) protocol, this is presumably the case because the finite-depth FL in experiment and the FL model converts a portion of the PWI model’s non-mixing regions into mixing regions by virtue of stretching in the FL^27^, a point we return to below.

For the half-full spherical tumbler mixed by alternating rotations about orthogonal axis, persistent non-mixing period-*n* regions always appear in pairs, which is a manifestation of the relationship between the full and half-iteration structures. For example, the two sets of non-mixing regions in Fig. 3b swap positions [Fig. 3d] upon an additional half-cycle of the mixing protocol. Thus, the B-set of non-mixing regions is the half-period offset of the A-set of non-mixing regions and vice versa.

Both PWI and FL models capture the gross features of the large scale persistent mixing and non-mixing structures observed in experiments for the two protocols we have examined so far. However, they do not describe transitions of the tracer particle into and out of the islands seen in experiments, e.g., Figs. 2b, 3a. This is because particle diffusion, which is driven by particle–particle collisions in granular flows, is not included in either model so that the underlying structure of the mixing is  more easily visualized. The collisional diffusion coefficient for flowing macroscopic particles scales as $d^2\dot \gamma$ where *d* is the diameter of the particles in the tumbler and $\dot \gamma$ is the shear rate^30^; in our tumbler and on average, diffusion randomly displaces a particle in the spanwise direction by ~d per FL pass^31^. For the (57°, 57°) protocol which produces ~1 FL pass per iteration, the root-mean-square displacement after 500-iterations is $d\sqrt {500} \approx 4.5$ cm, which is a bit more than half the tumbler radius. Based on the size of the non-mixing regions under the (57°, 57°) protocol, we expect a mean residency time of about 350 iterations in the regions, which is on the order of the observed residence times of at least 500 iterations in Fig. 2a, 326 iterations for the period-3 A regions in Fig. 2d, and 101 iterations for the period-3 B regions in Fig. 2d. Consequently, we expect particles in experiments to ‘leak’ out of non-mixing regions with a residence time that decreases as the square root of the size of the non-mixing regions. This is likely why smaller islands predicted by the FL and PWI models are not evident in the periodic orbits of the tracer particle in experiment. What is remarkable, however, is that even though diffusion is intrinsic in the experiments, it has minimal impact. Even with diffusion, the tracer particle remains in the non-mixing region [Fig. 2a] or returns to the non-mixing region after being bumped out of it by diffusion (Figs. 2b and 3b]. Thus, the fundamental structure of the flow (non-mixing regions predicted by the FL model or the PWI) persists in spite of diffusion.

**Fig. 4:**
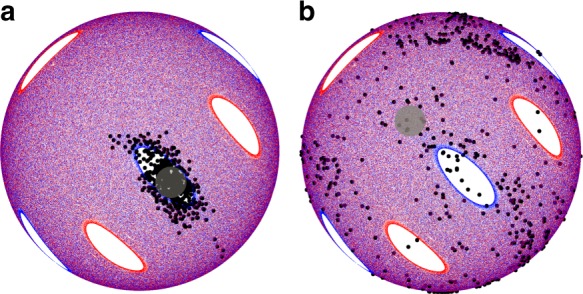
Influence of collisional diffusion. Comparison of the motion of 100 tracer particles under the (57°, 57°) protocol with collisional diffusion and initially located (gray circle) **a** within an island and **b** outside an island. After twelve iterations of the protocol, most of the particles initially located within the island (**a**) remain, while particles initially located outside the island (**b**) are distributed over the entire hemisphere due to chaotic mixing

To further illustrate the minimal influence of diffusion on particle motion, Fig. 4 shows the motion of 100 tracer particles starting within a gray circle inside and outside an island  after 12 iterations of the (57°, 57°) protocol. The tracers follow the advective flow field defined by the FL model but in addition undergo a random walk while in the FL with a mean step size per FL pass of *d*. After twelve iterations of the protocol, most of the tracers starting within the island are still within the island, while tracers starting outside the island are dispersed over the hemisphere. Thus, the chaotic mapping of tracer particles outside the island dominates the collisional diffusion. Similar results are obtained for tracer points with added diffusion in the PWI model.

**Fig. 5:**
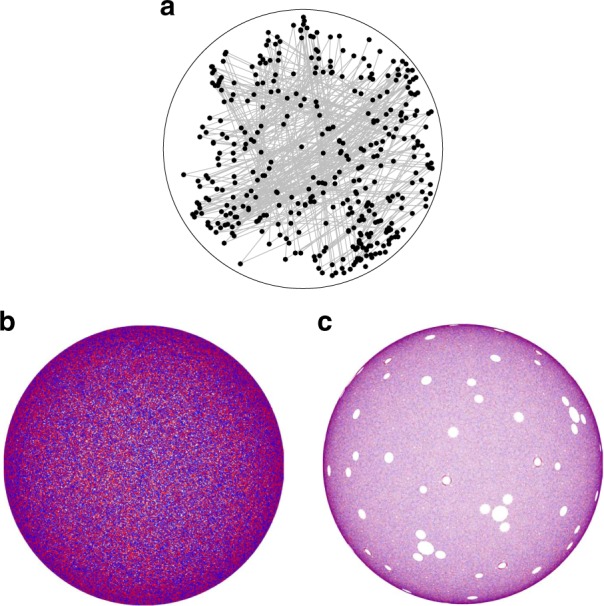
Mixing under the (75°, 60°) protocol. **a** Tracer particle positions (black points) and stroboscopic paths (gray lines) in a 500-iteration experiment continuously explore nearly the entire domain with no obvious periodic (non-mixing) regions. **b** In the corresponding FL model, tracers are mixed throughout the domain, consistent with the experiment. **c** The PWI model predicts a mostly mixed domain with a few small cells that are indiscernible in the experiment and FL model

Non-mixing islands in a background of mixing are the most common but not the only possibility; some protocols can produce nearly completely mixed domains. For example under the rotationally asymmetric (75°, 60°) protocol (Fig. 5), the tracer particle explores most of the domain in experiments [Fig. 5a], while the FL [Fig. 5b] and PWI [Fig. 5c] models generate mixing regions that completely and nearly completely, respectively, fill the domain. Note that complete mixing is not guaranteed by an asymmetric protocol, i.e., *θ*_*z*_ ≠ *θ*_*x*_, and, conversely, a symmetric protocol, i.e., *θ*_*z*_ = *θ*_*x*_, does not guarantee the existence of non-mixing regions.

### Barriers to mixing

**Fig. 6:**
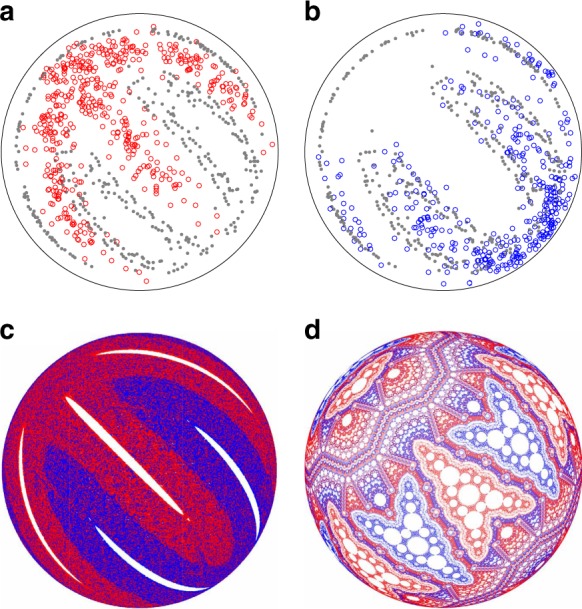
Period-3 mixing barrier between two halves of the domain for the (45°, 45°) protocol. **a**, **b** In 500-iteration experiments, the tracer particle (red and blue circles) cycles through two distinct parts of the domain every three periods depending upon its initial location. **c** The corresponding FL model has two thin sets of period-3 islands corresponding to the structure in experiment (**a**, **b**) [red islands in **c** correspond to red ‘fingers’ in **a** and blue islands in **c** correspond to blue ‘fingers’ in **b**]. The two sets of islands in experiment are separated by a mixing barrier (gray points in **a**, **b** which are extracted from the FL model). **d** The PWI model predicts two sets of period-3 arrowhead patterns

Under the three protocols used in Figs. 2–5, the non-mixing regions in experiments, also exist in the FL model and the PWI model (where they correspond to the largest cells). In contrast, under the (45°, 45°) protocol [Fig. 6], a mixing barrier emerges that divides the hemisphere into two regions and is not obviously captured by the PWI model. A tracer particle in two experiments [Fig. 6a, b] follows two different extended finger-like period-3 structures that are separated by a leaky barrier to particle transport and, together, cover the entire domain. These interdigitated period-3 structures are readily apparent in the FL model [Fig. 6c]. Red and blue tracers each dominate half of the domain with an elongated non-mixing region in each ‘finger,’ which roughly corresponds to the tracer particle positions from experiment. The region dominated by blue tracers appears to have only two ‘fingers.’ The third ‘finger’ is mostly contained in the FL, which is not visible in the view shown in Fig. 6c, though the blue edges of this ‘finger’ are evident at the periphery of the domain as well as the periphery in the experiments [Fig. 6b]. For the equivalent half-iteration structures in the FL model, the red tracer dominated regions alternate with the regions dominated by blue tracers with one of the red ‘fingers’ mapped to the FL. To delineate the two regions, we followed a tracer point in the FL model seeded between the red and blue tracer dominated regions to produce the gray points superimposed on the experimental data in Fig. 6a, b. The path of this tracer suggests a mixing barrier that wraps around the entire domain.

Unlike the PWI model structures for protocols shown in Figs. 2–5, the structure generated by the PWI model under the (45°, 45°) protocol [Fig. 6d] is less clearly related to the experiment and the FL model as it lacks the typical large cells that manifest as non-mixing regions in the experiment. Instead the PWI model generates large arrowhead-like features consisting of multiple small cells with transport barriers between adjacent red and blue arrowheads. However, the colors and positions of the arrowhead features correspond to the ‘finger’ features in the experiment and FL model.

### Dependence on flowing layer depth

**Fig. 7:**
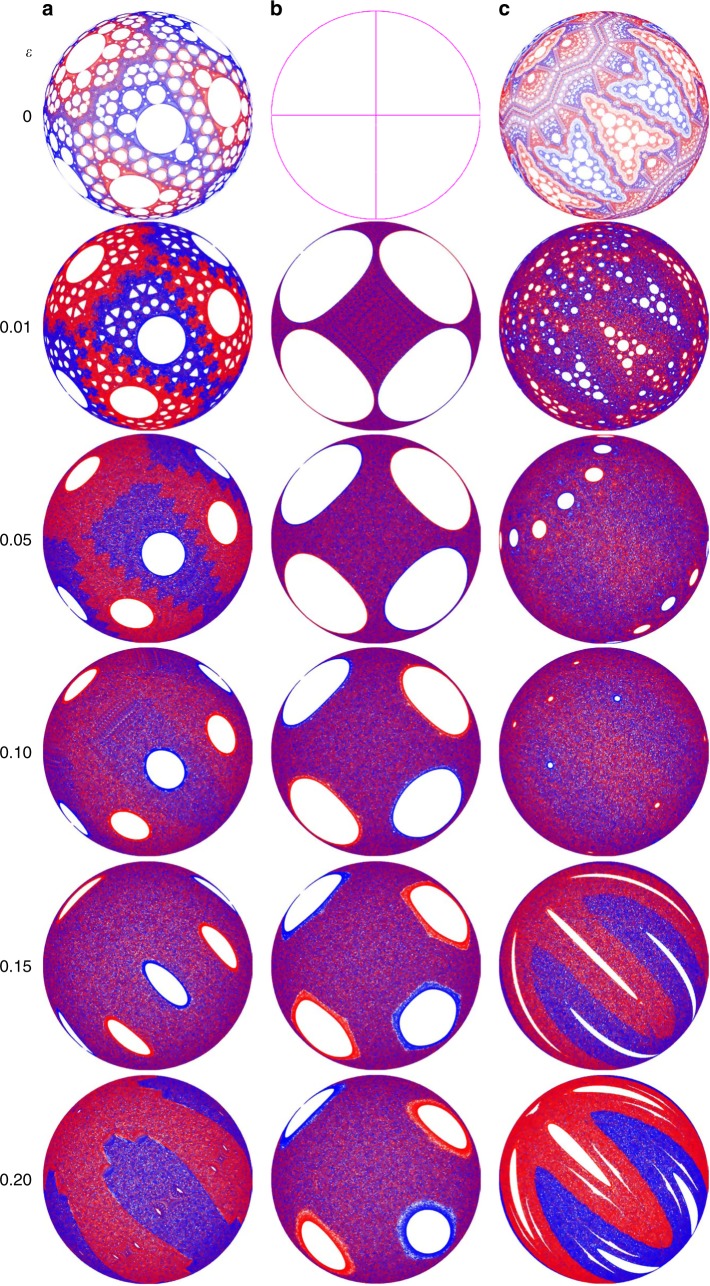
Effect of flowing layer depth on non-mixing regions. Three different protocols are examined (columns): **a** (57°, 57°), **b** (90°, 90°), and **c** (45°, 45°). With increasing flowing layer depth, $\epsilon$ (rows), non-mixing regions formed by cutting-and-shuffling ($\epsilon$ = 0) shrink in **a**–**c** and disappear in **a**, **c**, while new non-mixing regions originating from stretching-and-folding in the finite depth flowing layer appear and grow in **c** for $\epsilon$ ≥ 0.15

The structures generated by the FL model match experimental observations regardless of protocol, while those predicted by the PWI model are not always evident in experiment or the FL model. For example, the small non-mixing cells in the PWI model for the four protocols we examine above are missing in results from experiments and the FL model. As explained above, lack of fine structure is expected in experiments where granular diffusion is present, but this does not explain their absence from the diffusion-less FL model. To better understand the relationship between the non-mixing structures in the PWI and FL models, particularly for the (45°, 45°) protocol, we consider the influence of the FL depth. Physically, the FL depth increases with rotation rate *ω* and decreases with shear rate $\dot \gamma$, since $\epsilon = \sqrt {\omega {\mathrm{/}}\dot \gamma }$ = *δ*(0, 0)/*R*^32^, and is typically ~10 particle diameters in experiments. As Fig. 7 shows, non-mixing regions shrink and, in most cases, vanish with increasing FL depth in the FL model. Nearly all cells present in the PWI model (i.e., $\epsilon$ = 0) disappear under the (57°, 57°) and (45°, 45°) protocols at $\epsilon$ = 0.2 and $\epsilon$ = 0.1, respectively, while under the (90°, 90°) protocol, cells shrink but persist up to the largest $\epsilon$ examined.

**Fig. 8:**
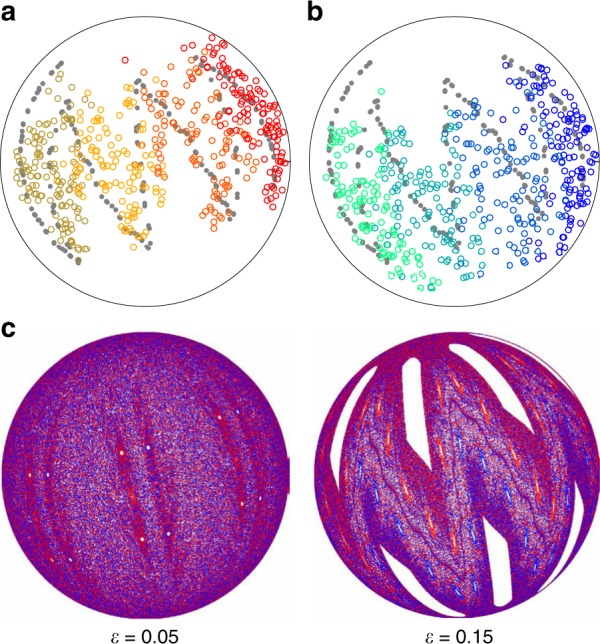
Weak elliptic non-mixing barrier around period-4 regions under the (45°, 15°) protocol. **a**, **b** The tracer particle in 500-iteration experiments only occasionally crosses the mixing barrier (gray points). Color map illustrates the period-4 cycle. **c** Non-mixing regions are not evident for $\epsilon$ = 0.05 but become prominent with increasing flowing layer depth in the FL model

Focusing on the (45°, 45°) protocol [Fig. 7c], the cells present at $\epsilon$ = 0 shrink and eventually annihilate with increasing $\epsilon$. However, new structures emerge for larger $\epsilon$ characterized by both non-mixing regions and a mixing barrier between red and blue tracer particles. In particular, at $\epsilon$ = 0.15, the leftmost red ‘finger’ and its central non-mixing region land in the FL after a half-iteration, while each of the subsequent red ‘fingers’ map to the FL one iteration apart. The same phenomenon occurs for the blue ‘fingers’ except on full iterations. The FL acts as a mixing barrier in the (45°, 45°) protocol as each part of the fixed bed lands in the FL during a change in the rotation axis. This behavior is not unique to the (45°, 45°) protocol as Fig. 8 illustrates for the (45°, 15°) protocol. Here, the experiments show evidence for two sets of period-4 islands separated by a mixing barrier. Again, the FL model shows that these features emerge for finite $\epsilon$

.

### Motion of persistent vs. emergent non-mixing regions

**Fig. 9:**
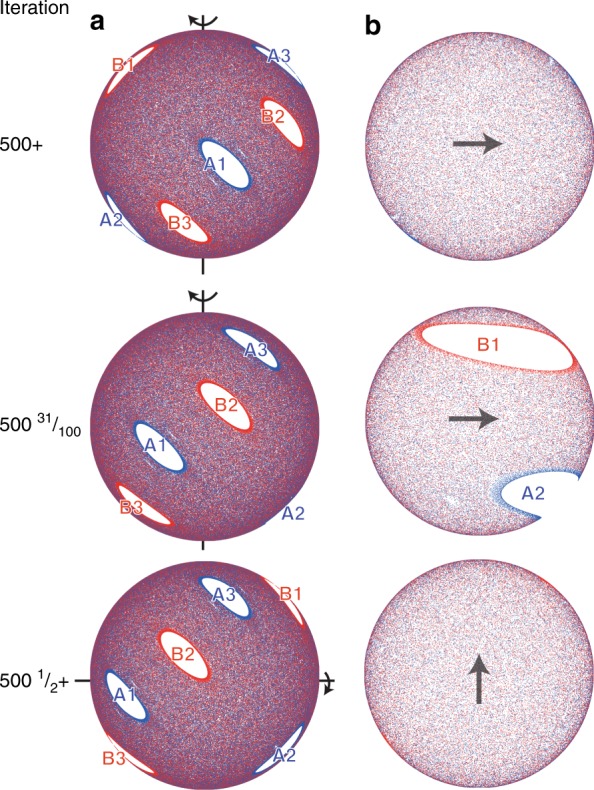
Movement of persistent non-mixing regions through the flowing layer. Non-mixing regions (A1-A3, B1-B3) present at $\epsilon$ = 0 [see Fig. 7a] are still present at $\epsilon$ = 0.15 and pass fully through the flowing layer after each half-iteration [(57°, 57°) protocol]. Views from below of **a** the hemispherical shell and **b** the planar flowing layer. Labels of periodic regions illustrate changes in orientation and arrows indicate the direction of flow in the flowing layer. Animations are provided in Supplementary Movie 1

**Fig. 10:**
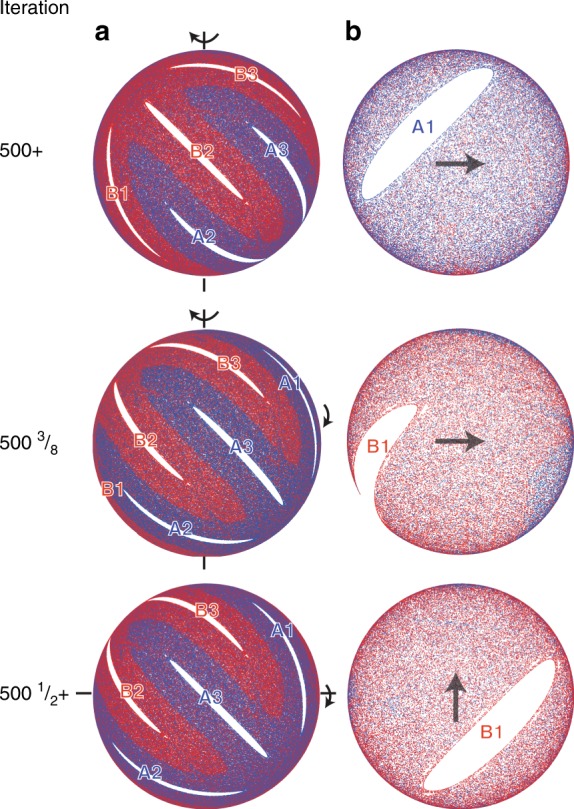
Movement of emergent non-mixing regions through the flowing layer. Emergent non-mixing regions (A1-A3, B1-B3) appear only for $\epsilon$ > 0 (i.e., non-existent in the PWI model) and their accompanying ‘fingers’ alternate between being fully contained in the flowing layer or in the bulk after each half-iteration for the (45°, 45°) protocol. Views from below of **a** the hemispherical shell and **b** the planar flowing layer for $\epsilon$ = 0.15. Labels of periodic regions illustrate changes in orientation. Animations are provided in Supplementary Movie 2. Arrows in **b** indicate the direction of flow in the flowing layer

The primary difference between non-mixing regions present at $\epsilon$ = 0 that persist for $\epsilon$ > 0 and non-mixing regions that emerge only for $\epsilon$ > 0 is that the persistent regions completely exit the FL at the end of a rotation while the emergent regions are always fully contained within the FL at the end of a rotation. This difference is illustrated in Figs. 9, 10 (and in Supplementary Movies 1 and 2, respectively) which depict the usual images of the hemispherical bottom surface viewed from below, plus images of the free surface of the FL viewed from below in order to visualize the passage of the non-mixing region through the FL. In addition to images at the full iteration (500+), these figures also include images of both surfaces during and just after completing the first rotation of the 501st protocol (i.e., 500${\textstyle{1 \over 2}}$+).

Viewed in this way, Fig. 9 reveals two generic features of persistent non-mixing regions [illustrated here for the (57°, 57°) protocol]. First, persistent non-mixing regions pass entirely through the FL during each rotation, regardless of the FL depth, which rearranges (‘shuffles’) them on the hemisphere. Second, their boundaries are set by the boundary of the FL during the interchange of rotation axes (the ‘cut’). This is particularly evident in Supplementary Movie 1, but can also be seen in Fig. 9. At 500+ iterations, non-mixing regions A2 and A3 are just touching the circular edge of the FL. Between iteration 500+ and 500${\textstyle{1 \over 2}}$, corresponding to the first 57° rotation of the iteration, non-mixing regions A2 and B1 are stretched as they pass through the FL. At 500${\textstyle{{31} \over {100}}}$ the FL image shows B1 stretched completely across the the FL while only the trailing portion of A2 is visible as it finishes crossing the FL. In the subsequent half-iteration, A2 and B3 pass through the FL in the direction of the arrow.

Returning to the case of emergent structures that appear only for $\epsilon$ > 0, Fig. 10 illustrates [using the (45°, 45°) protocol] that their motion is quite different from that of persistent islands. First, emergent islands periodically land entirely within the FL at the end of a rotation and are stretched in the streamwise direction. Subsequent rotation about the orthogonal axis stretches them in the opposite direction before they return to the bulk. Second, their boundaries are not necessarily coincident with the FL boundary at the end of a rotation. Third, the boundary of the mixing barrier (between red and blue ‘fingers’) maps approximately to the boundary of the FL as shown by the mostly blue FL at 500+ iterations and the mostly red FL at 500${\textstyle{1 \over 2}}$+ iterations. However, the mixing barrier between red and blue regions is less clearly defined than the white non-mixing region boundaries.

## Discussion

Cutting-and-shuffling offers an expanded new framework from which to consider mixing, an approach that we have only begun to investigate. Based on this study of mixing in a model system consisting of a spherical tumbler under a biaxial mixing protocol, we show that the mathematics of piecewise isometries (the PWI model) can accurately predict the mixing and non-mixing regions observed in experiments when the FL depth is sufficiently small. While the mathematical limit of cutting-and-shuffling represented by the PWI model provides a skeleton of the structure of solid-like mixing, fluid-like stretching in the FL modifies that structure. The continuum FL model connects the idealized non-mixing regions evident in the PWI model with the modified non-mixing regions observed in experiments, though without collisional diffusion. In our model system, a deeper FL increases the fraction of particles in the FL and the time they spend there, which then increases the influence of mixing via stretching-and-folding. We expect other 3D dynamical systems with coexisting solid and fluid regions (which include many practical mixing devices such as bladed mixers and plows in addition to tumblers) to exhibit similar complex mixing structures that result from the distinct and competing mixing processes associated with fluid stretching-and-folding and solid cutting-and-shuffling^27^. A deeper understanding of these ‘hybrid-mixing’ systems based on the structures determined by PWI theory is expected when the spatial extent of flowing regions is limited or when the flows are weakly shearing.

Finally, the cutting-and-shuffling paradigm for mixing lies at the intersection between the abstract mathematical theory of piecewise isometries and dynamical systems approaches to mixing. While we have shown that non-mixing regions occur under certain conditions, it may be possible to improve both the degree of mixing and the rate of mixing by varying the cut-and-shuffle parameters at each iteration using optimal control strategies to develop ‘time-dependent’ cut-and-shuffle mixing strategies. Of course, compared to smooth dynamical systems, cutting-and-shuffling uses discrete time steps and generates complex, multi-modal, discontinuous distributions of mixing metrics across the parameter space, all of which present new challenges in optimal control. Furthermore, the cuts can occur anywhere, so the combinatoric methods used to study discrete space shuffling do not necessarily apply. Nevertheless, combined cutting-and-shuffling and stretching-and-folding strategies offer the potential to actively control the degree and rate of mixing, which may be significant in many physical systems.

## Methods

### Experiments

Experiments were conducted using *d* = 1.89 ± 0.09 mm diameter soda-lime glass beads (SiLiglit Deco Beads, Sigmund Lindner GmbH, Germany) in a half-filled *D* = 14 cm diameter acrylic spherical tumbler mounted on an apparatus that performs the biaxial rotation protocol and can perform X-ray imaging, see schematic in Supplementary Fig. 1. A density-matched (*ρ* = 2.5 g cm^−1^) *d*_tracer_ = 4 mm diameter X-ray opaque tracer particle constructed from two 3D-printed plastic hemispherical shells with a Pb-Sn solder sphere in the center was used to visualize the flow. The larger diameter of the tracer particle caused the particle to flow at the surface of the FL and near the tumbler wall in the solid bed [outermost path in Supplementary Fig. 1a]. Components of the spherical tumbler apparatus within the X-ray beam path consist of X-ray transparent materials (aluminum and plastic) to ensure an image suitable for particle tracking.

For each action (**U** or **W**) of the BST (see Supplementary Fig. 2) protocol, the sphere was rotated about a single axis for protocol angle (*θ*_*z*_ or *θ*_*x*_) by three wheels driven by a motor mounted on a turntable at rotation speed *ω* = 2.6 rpm, see Supplementary Fig. 1b. Since an initially static particle bed only starts to flow when tilted beyond its static angle of repose *β*_*s*_ with respect to the horizontal, the sphere was slowly rotated so that the free surface was just below *β*_*s*_ prior to each action. Then, when a protocol action (rotation) was initiated, there was a small avalanche at the start of flow as the free surface relaxed to the dynamic angle of repose *β* < *β*_*s*_, after which the flow was continuous. After each action, the tumbler was rotated in the reverse direction to ensure the free surface was horizontal before switching the rotation axis. The apparatus reoriented the drive wheels for the next rotation by raising the tumbler off the wheels, rotating the turntable to make the wheel axes parallel to the next tumbling axis, and then lowering the tumbler back down onto the wheels. To minimize accumulated error from executing the protocol over many iterations, a position sensor was used to ensure that the turntable returned to the original axis after each iteration. Additionally, a thin X-ray opaque fiducial marker (lead tape) was mounted on the interior wall of the spherical tumbler to track any tumbling deviations. With these measures in place, the system has an angular displacement error of less than 1° per protocol iteration. The errors are not systematic and, hence, average to zero.

A digital video camera (Point Grey BlackFly 12A2M) mounted directly beneath the output image port of the X-ray image intensifier [see Supplementary Fig. 1a] provides a subsurface bottom view of the tumbler. Images were acquired prior to each tumbling action: the first frame, when the free surface was horizontal and subsequent frames while the tumbler was slowly rotating to the angle of repose. The direction and magnitude of tracer particle displacement in these frames identified whether the particle was at the free surface or in the bulk near the tumbler wall. Image distortion due to image intensifier and camera optics was corrected using the MATLAB Image Processing Toolbox function $\tt{imwarp}$. The geometric transformation required by the function $\tt{imwarp}$ was a 4th order polynomial model generated from applying the function $\tt{fitgeotrans}$ to an image obtained from a Cartesian hole pattern (diameter 3.5 mm with 5.1 mm spacing). The corrected hole pattern image was used to generate the matrix transformation from the hole pattern’s pixel spacing to the physical grid dimensions. To track the tracer particle and the fiducial indicator, each image was divided by a background image generated from the average of all iterations from a particular run to reveal the two features of interest. The tracer particle and the fiducial indicator were distinguishable from each other by their eccentricity and were tracked automatically using 2D feature finding MATLAB algorithms developed by the Kilfoil group (Code repository hosted at http://people.umass.edu/kilfoil/tools.php) using methods from Crocker et al.^33^.

### Flowing layer model

The FL model^9,34^ assumes that the flow is primarily two-dimensional in the streamwise direction and confined to a thin lenticular FL with a constant depthwise shear rate $\dot \gamma$ for each tumbling action **U** and **W**, see Supplementary Fig. 2. Using a Cartesian coordinate system with origin at the center of the radius *R* spherical tumbler, rotation is clockwise about the *z*-axis and *x*-axis at a rotation speed *ω* for an angular displacement *θ*_*z*_ and *θ*_*x*_ for **U** and **W** actions, respectively. For convenience, all variables are dimensionless—length scales (*x*, *y*, *z*, *r*, and FL depth *δ*) are normalized by *R* and rotation period *T* is normalized by 1/*ω*. The interface between the FL and the bulk is given by *δ*(*x*, *z*) = $\epsilon \sqrt {1 - x^2 - z^2}$, where $\epsilon = \sqrt {\omega {\mathrm{/}}\dot \gamma }$ is the maximal dimensionless FL depth (at the center *x* = *z* = 0). The velocity **u** = (*u*, *v*, *w*) is piecewise defined for the FL and the bulk. For the **U** action, the FL (*y* ≥ −*δ*) velocity is **u**_fl_ = $((\delta + y){\mathrm{/}}\epsilon ^2,xy{\mathrm{/}}\delta ,0)$ and the bulk (*y* < −*δ*) is in solid body rotation with velocity profile **u**_b_ = (*y*, −*x*, 0). Analogously, the velocity field for the **W** action is obtained by interchanging *x*- and *z*-components.

Particle positions are generated by integrating the velocity field using the Runge-Kutta (RK4) method in the FL and the semi-implicit Euler method in the bulk^9,34^. The dimensionless time step is Δ*t*/*T* = 5 × 10^−5^ to ensure stability. A FL depth at the midpoint of the FL, *δ*(0, 0), of $\epsilon$ = 0.15 matches the experiments (*ω* = 2.6 rpm) based on the tracer particle FL passage time. To investigate the impact of the FL depth, conditions with $0 \le \epsilon \le 0.20$ were also simulated. For context, previous studies on quasi-2D flows reported FL depth of $\epsilon \approx 0.1$^32^, depending on the particle to tumbler diameter ratio *d*/*D*.

Stroboscopic maps (also known as Poincaré sections or discrete time maps) of 500 iterations were used to investigate mixing and non-mixing behavior and to provide direct comparison to experiments. The stroboscopic maps utilized tracers seeded at the intersection of the FL boundary and the *r* = 0.9 hemispherical shell at zero iterations (blue points) and at the first half-iteration (red points). Regions avoided by the tracer particles correspond to elliptic regions (islands). The outer boundary orbits of these elliptic regions were obtained by finely seeding initial conditions near the boundary in successive stroboscopic maps until the boundary orbits were extracted. The periodicity of these elliptic regions was determined by tracking tracers seeded in these regions. Other larger elliptic orbits that wrap around the bulk and the FL (e.g., gray points in Figs. 6a, b and 8) were extracted by seeding a tracer in the neighborhood of the orbit.

### Piecewise isometry model

The PWI model is applicable in the infinitely thin FL (ITFL) limit ($\epsilon$ = 0) of the FL model^26,35,36^. Like the continuum model, stroboscopic maps were used to investigate mixing and non-mixing regions. Because the flow dynamics are radially invariant with an ITFL, tracer positions are mapped by their angular displacements on the hemispherical shell. When tracers reach the free surface, they are instantaneously reflected across the ITFL in the streamwise direction. The initial positions of the passive tracers in the stroboscopic maps were selected to lie along the isometry partitions at zero iterations (blue points in figures) and the first half-iteration (red points in figures) and mapped for 10,000 iterations to assure convergence.

### Data availability

The data that support the findings of this study are available from the corresponding author upon reasonable request.

### Code availability

The computer codes used in this study are available from the corresponding author upon request.

## Electronic supplementary material

Supplementary Information

Description of Additional Supplementary Files

Supplementary Movie 1

Supplementary Movie 2
