# Supplementary material for: Persistent structures in a three-dimensional dynamical system with flowing and non-flowing regions
Source: Nat Commun. 2018 Aug 7;9:3122. doi: 10.1038/s41467-018-05508-7 (PMC6081420; doi:10.1038/s41467-018-05508-7)
Supplement: Supplementary file 1 — Supplementary Information [file 41467_2018_5508_MOESM1_ESM.pdf]

Persistent structures in a three-dimensional dynamical system with flowing and non-flowing regions

Zaman et al.

Supplementary Information

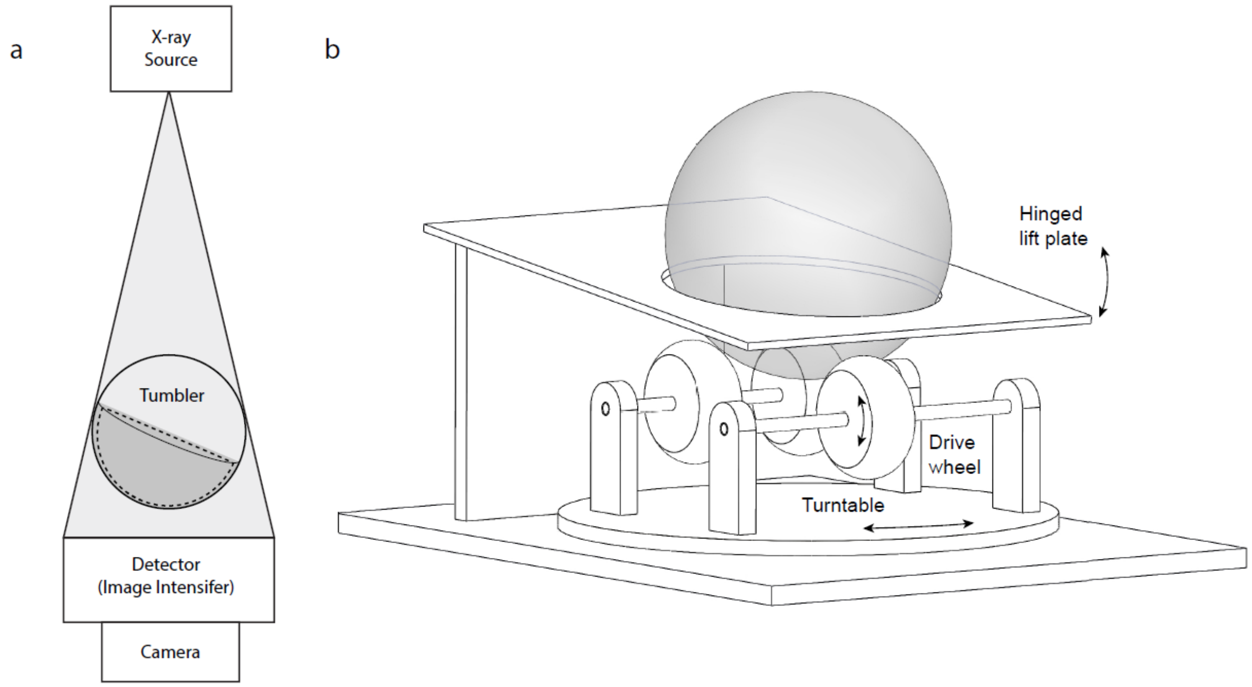

Supplementary Figure 1: **Experimental apparatus.** (a) Front view cross-section of the tumbler system. X-ray source projects a vertical cone beam over the spherical tumbler and X-ray image intensifier equipped with a camera for imaging. Vertical cross section of the half full tumbler shows the bed (dark gray region) with a thin lenticular flowing layer. The larger density matched X-ray opaque tracer particle path (dashed curve) is in solid body rotation close to the tumbler wall in the bed and flows along the free surface of the flowing layer. (b) Schematic of apparatus used to realize the biaxial spherical tumbling protocol in experiments. The rotation axis is switched by lifting the tumbler and rotating the turntable supporting the drive and support wheels to a new orientation.

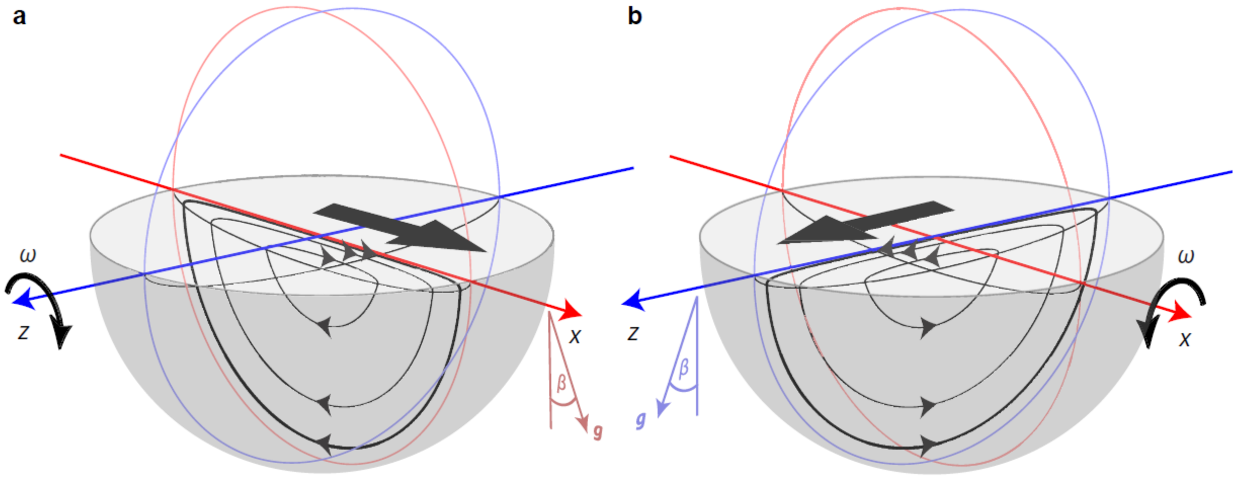

Supplementary Figure 2: **Biaxial spherical tumbler flow**. The flow consists of an alternating sequence of two single-axis actions (a) **U** and (b) **W** about the *z*- and *x*-axis, respectively, at rotation speed ω. During each action, particles form a thin lenticular flowing layer that flows in the direction normal to the rotation axis (large flat black arrow near the center of the tumbler). Particles from the downstream half of the flowing layer are deposited onto the solid bed of particles that rotates with the tumbler wall, and they remain there until they reach the flowing layer again. The angle of the flowing free surface is β with respect to gravity, *g*. Three mean particle paths are shown for a two-dimensional slice for each action, with the bold path (near the free surface and closest to tumbler wall) being a representative path for the large X-ray opaque tracer particle used in the experiments.
