# Supplementary material for: Persistent structures in a three-dimensional dynamical system with flowing and non-flowing regions
Source: Nat Commun. 2018 Aug 7;9:3122. doi: 10.1038/s41467-018-05508-7 (PMC6081420; doi:10.1038/s41467-018-05508-7)
Supplement: Supplementary file 2 — Description of Additional Supplementary Files [file 41467_2018_5508_MOESM2_ESM.pdf]

Supplementary Movie 1: Motion of persistent non-mixing regions under the (57°, 57°) protocol. Bottom views of (a) the bed and (b) the flowing layer for finite flowing layer thickness (ε= 0.15). During each rotation, a pair of blue and red islands passes through the flowing layer and reappears in the bed on the other side after rotation completes. No islands are present in the flowing layer when the rotation axis is switched. The initial condition in the video is generated by tracking the positions of particles, initially seeded on the edge of the flowing layer before the first and second rotations, for 500 iterations. Particles are advected for the next three full iterations in the video.

Supplementary Movie 2: Motion of emergent non-mixing regions under the (45°, 45°) protocol. Bottom views of (a) the bed and (b) the flowing layer for finite flowing layer thickness (ε= 0.15). The three blue islands appear sequentially in the flowing layer at each full-iteration and return to their original locations after three iterations. Identical behavior is demonstrated by red islands, but on half-iterations. The initial condition in the video is generated by tracking the positions of particles, initially seeded on the edge of the flowing layer before the first and second rotations, for 500 iterations. Particles are advected for the next three full iterations in the video.
